# Evaluation of Alvarado score in diagnosing acute appendicitis

**DOI:** 10.11604/pamj.2019.34.15.17803

**Published:** 2019-09-06

**Authors:** Mahmoud Musa Al Awayshih, Mohammad Nabih Nofal, Ali Jad Yousef

**Affiliations:** 1 General Surgery and Anesthesia Department, College of Medicine, Mutah University, Karak, Jordan

**Keywords:** Acute appendicitis, scoring system, Alvarado score

## Abstract

Using a practical scoring system for diagnosing acute appendicitis can help reduce the rate of unnecessary surgery. This prospective study was carried out to evaluate Alvarado scoring system for diagnosing of acute appendicitis in our set up. Out of total 100 patients, appendicitis was confirmed in 80 patients, thus giving negative appendectomy rate of 20% (male 6%, female 14%). Perforation rate was 4%. Positive predictive value was 89%. The sensitivity was 54% and specificity 75%. Alvarado score is not a sensitive tool for aiding diagnosis of acute appendicitis.

## Introduction

Acute appendicitis is the most common abdominal surgical emergency with a lifetime cumulative incidence of 7% [1]. In 1886; Fitz described the traditional signs and symptoms of acute appendicitis [2]. The diagnosis of acute appendicitis is mainly clinical based on history, clinical examination and sometimes aided by laboratory investigations (such as white blood cells count or CRP level). Imaging modalities are not requested routinely because they have been shown to add very little information unless there are complications. The definitive diagnosis is achieved at surgery and after histopathologic examination of the resected appendix [3]. Delay in diagnosis and management may result in significant morbidity. A number of scoring systems have been developed for aiding in the early diagnosis of acute appendicitis. Scoring systems are a valuable and valid instrument of discrimination between acute appendicitis and nonspecific abdominal pain [4]. Alvarado scoring system, which was introduced in1986, is one of these systems and is based on history, clinical examination and few laboratory findings (Table 1) [5].

**Table 1:** Alvarado scoring system

Symptoms	Score
Migratory right iliac fossa pain	1
Nausea/ vomiting	1
Anorexia	1
**Signs**	
Tenderness in right iliac fossa	2
Rebound tenderness in right iliac fossa	1
Elevated temperature	1
**Laboratory Findings**	
Leukocytosis	2
Shift to the left of neutrophils	1
Total	10

## Methods

This study was carried out on 100 consecutive patients admitted to the Surgical Ward of Karak Teaching Hospital, Karak-Jordan, from the emergency department with the clinical diagnosis of acute appendicitis during the period from April 2013 to December 2014. Patients of any age group and both sexes presenting to the emergency department with pain in right iliac fossa pain and operated with appendectomy were included in the study. Patients with clear presentation of other diagnoses such as urological, gynecological or acute abdomen conditions other than appendicitis were excluded from the study. All included patients were admitted after initial assessment in the emergency department and base-line investigations including complete blood count, urine routine examination, and serum bilirubin were done. Then study format was filled in for each patient by a general surgery officer. The format had general information about the patient demographics in addition to Alvarado score variables. The sums of all the scores were calculated for each patient and according to the results patients were divided into two groups: group I Alvarado score ≤ 7 (low and intermediate suspicion group), and group II Alvarado score ≥ 7 (high suspicion group). All the patients underwent appendectomy on clinical bases after variable hours of observation, and the surgical specimens were examined grossly and pathologically. The formats were completed with pathology reports and post operative course. Finally, the sensitivity, specificity and positive predictive value (the proportion of patients with a positive test result who actually have the disease) of Alvarado scoring system were calculated negative appendectomy rate.

## Results

Our study was conducted on one hundred consecutive patients with clinical picture of acute appendicitis. Among these patients 44 were female (44%) and 56 were male (56%). The male to female ratio was 1:1.2. Mean age was 22.9 years (range 5-61 years, standard deviation +12.5 years), with median age of 19 years. The frequency distribution of patients according to Alvarado scoring system is shown in Table 2. In group I we had 52 patients totally: 2 patients (2%) with Alvarado score of 3-5, one female and one male, and both had unremarkable appendix pathology. 50 patients (50%) with Alvarado score of 5-7, 25 males and 25 females: the appendix was perforated in two patients, a 40-year old female and an 8- year old male; gangrenous in one patient: a 13-year old female, and unremarkable in 13 patients: ten females and 3 males. The remaining cases were mostly purulent appendicitis. The true negative (normal appendix) for group I is, thus, 15 cases. In group II; 48 patients the score was 7-9: 30 males, and 18 females: the appendix was perforated in two male patients, and unremarkable in five patients (the false positive), the remaining was mostly gangrenous acute appendicitis. The appendix was normal or unremarkable in 20 (14 females, six males) patients which means a 20% negative appendectomy rate, and perforated in four (three males, one female) patients; perforation rate 4%. The pathology results are shown in Table 3. In this study, the sensitivity of Alvarado score was 54%, specificity 75%, and positive predictive value 90% as shown in Table 4.

**Table 2:** Alvarado score distribution among patients

Alvarado Score	n
3-5	2
5-7	50
7-9	48
Total	100

**Table 3:** Pathology types in 100 appendix specimens

Type	Number of case
Gangrenous	30
Purulent	37
Perforated	4
Catarrhal	9
Unremarkable	20

**Table 4:** Diagnostic accuracy of Alvarado score

Group	n	Confirmed Appendicitis	Normal Appendix
I (Alvarado score ≥ 7)	48	43 (True positive)	5 ( False positive)
II (Alvarado score≤ 7)	52	37 (False negative)	15 (True negative)

Sensitivity 54% specificity 75% positive predictive value 90%

## Discussion

In spite of being the most common cause of acute abdomen, acute appendicitis remains a challenging diagnosis because it is basically a clinical diagnosis which has many clinical pictures. The negative appendectomy rate in this series was 20% which is congruent with the rates reported in the literature of 8 to 33% [6]. Clinical scoring systems proved to be useful in the diagnosis of some surgical conditions. In the past few years deferent scoring systems have been developed to help the diagnosis of acute appendicitis [7]. Although, many scoring systems have been advocated but most are sophisticated and hard to implement in the real clinical situation [7]. On the other hand, Alvarado scoring system is a simple system that can be used easily in the clinic or emergency department [5]. To be helpful, the scoring system must be both sensitive and specific. The sensitivity in this study was 54% which is similar to that reported by Al Hashemy and Seleem [6] and lower than that reported by Lone *et al* of 88% [8]. The positive predicative value 90% in this study is close to that of other studies [9,10]. Our study shows that Alvarado scoring system is not sensitive enough to be helpful in the diagnosis of acute appendicitis. In addition, it may not be accurate in diagnosing acute appendicitis even in patients with score ≥ 7 as shown in our series with 5 false positive cases out of 48 (10%).

## Conclusion

In this study, Alvarado scoring system was not found to be a very helpful system in aiding the diagnosis of acute appendicitis in our setup, probably due to the heterogeneous population studied. Further refinement and adjustments may be needed to improve sensitivity of the scoring system and decrease the controversy over its use routinely.

### What is known about this topic

- The diagnosis of acute appendicitis is basically clinical;
- A diagnostic scoring system will enhance correct diagnosis;
- Correct diagnosis will reduce rate of unnecessary surgery.

### What this study adds

- Alvarado score is not a sensitive scoring system for diagnosing acute appendicitis;
- Clinical judgment cannot be overemphasized for diagnosing acute appendicitis;
- Should there be an effective scoring system, it should be refined according to patient’s stratification.

## Competing interests

The authors declare no competing interests.
